# VAMP7j: A Splice Variant of Human VAMP7 That Modulates Neurite Outgrowth by Regulating L1CAM Transport to the Plasma Membrane

**DOI:** 10.3390/ijms242417326

**Published:** 2023-12-10

**Authors:** Matteo Gasparotto, Elena Dall’Ara, Marcella Vacca, Francesco Filippini

**Affiliations:** 1 Synthetic Biology and Biotechnology Unit, Department of Biology, University of Padua, Via U. Bassi 58/B, 35131 Padova, Italy; matteo.gasparotto.1@phd.unipd.it (M.G.); elena.dallara@studenti.unipd.it (E.D.); 2 Institute of Genetics and Biophysics “A. Buzzati Traverso”, Consiglio Nazionale delle Ricerche (CNR), Via Pietro Castellino, 111, 80131 Naples, Italy

**Keywords:** subcellular trafficking, longin domain, VAMP7, alternative splicing, evolutionary cell biology, neurodevelopment, neurite outgrowth, L1CAM, tyrosine phosphorylation

## Abstract

The vesicle-associated membrane protein 7 (VAMP7) is a SNARE protein of the longin family involved in a wide range of subcellular trafficking events, including neurite sprouting and elongation. The expression of the human gene *SYBL1*, encoding VAMP7, is finely regulated by alternative splicing. Among the minor isoforms identified so far, VAMP7j is the one most expressed and modulated in the human brain. Therefore, we focused on gaining functional evidence on VAMP7j, which lacks a functional SNARE motif but retains both the longin and transmembrane domains. In human SH-SY5Y cells, we found VAMP7j to modulate neuritogenesis by mediating transport of L1CAM toward the plasma membrane, in a fashion regulated by phosphorylation of the longin domain. VAMP7-mediated regulation of L1CAM trafficking seems at least to differentiate humans from rats, with VAMP7j CNS expression being restricted to primates, including humans. Since L1CAM is a central player in neuritogenesis and axon guidance, these findings suggest the species-specific splicing of *SYBL1* is among the fine tuners of human neurodevelopmental complexity.

## 1. Introduction

A challenging task in current biology is the search for elements and mechanisms mediating those higher cognitive and behavioral abilities that “make us human”. In the absence of evident massive genomic change, species-specific finetuning, at multiple regulatory layers, is likely to provide human neuronal differentiation and neurodevelopment with increased functional complexity.

The subcellular trafficking (SCT) machinery mediates crucial events in early neuronal differentiation, as it drives the shaping of the highly polarized neuronal cells, followed by outgrowth and elongation of their processes. Indeed, the sprouting and elongation of neurites require plasma membrane (PM) expansion, the insertion of new protein components, and the repositioning of already resident molecules [1,2].

Along the whole neurodevelopment process, SCT routes also determine the sorting to the PM of attractive and repulsive extracellular guidance cues, which in turn indicate axons how to properly connect with each other in building up a functional neural network. Such cues are sensed with impressive accuracy by the growth cone, a specialized structure on the tip of the growing neurite [3], prompting neurons to establish proper connections during the shaping and reshaping of the developing neural network.

In particular, neuronal cell adhesion molecules (CAMs) with immunoglobulin (Ig)-like repeats are critical in orchestrating the growth cone functionality and developmental signals via homo- and heterophilic interactions [4,5]. In several neuronal CAM families, these interactions depend on a conserved neurite outgrowth and guidance (NOG) motif at their Ig2 repeat [6]. These proteins are prototyped by L1CAM, which is known to play a pivotal role in neuronal differentiation and when mutated may cause severe neurodevelopmental disorders [7]. Trans-homophilic binding between L1CAM molecules on opposing cells (or between cells and the extracellular matrix, ECM) is a known trigger for neurite growth [5]. Therefore, the SCT routes that regulate the proper sorting of L1CAM are central to both neuritogenesis and neurodevelopment. L1CAM transport toward the PM is mediated by vesicle-associated membrane protein 7 (VAMP7) in the developing rat brain and primary neurons, as well as in rat-derived PC12 cells [8].

VAMP7, Sec22b, and Ykt6 are prototypes for the three main subfamilies of longin family proteins (or longins), which are conserved in all eukaryotes [9]. Longins are characterized by an N-terminal longin domain (LD) [10], followed by a soluble N-ethylmaleimide-sensitive factor attachment protein receptor (SNARE) motif and a membrane anchor, which in VAMP7 and Sec22b consists of a transmembrane domain (TMD) with a cytoplasmic tail [9]. The longin domain is a building block of the eukaryotic SCT machinery, where it represents the most conserved and widespread domain, as proteins consisting of (or embedded with) an LD are found in all SCT routes and complexes [11]. In longins, the LD modulates the SNARE motif availability to participate in the *trans*-SNARE complex by intramolecular binding to the N-terminal half of the SNARE motif [12,13]. In human VAMP7, phosphorylation of tyrosine 45 of the LD inhibits the formation of such closed conformation, thus favoring the SNARE-mediated membrane fusion [14]. Interactions of the LD with different complexes determine the longins’ subcellular localization (SCL) [12,15]. The binding of the VPS9-domain and ankyrin repeat containing protein (VARP), a Rab32/38 effector and kinesin 1-interacting protein, to the VAMP7 closed conformation, favors its transport towards the PM [16,17].

Longins Sec22b and Ykt6 are involved in the ER-to-Golgi transport [9], while VAMP7 is involved in a wider range of SCT events, e.g., late endosome–lysosome fusion, autophagosome biosynthesis, autophagosome–lysosome fusion, autophagosomal secretion, mitosis, cell migration, membrane repair and growth, and membrane remodeling in neuritogenesis [16,18,19,20,21,22]. In land plants, VAMP7 shows gene amplification and gives rise to a fourth, evolutionarily restricted subfamily of non-SNARE longins, named phytolongins [23]. These proteins share a similar domain topology with VAMP7 but, in-between the LD and TMD, a non-SNARE region of unknown function replaces the central SNARE motif. Non-SNARE longins are found in animals as well, where Sec22a and Sec22c paralogs [24] share the LD and membrane anchor with Sec22b, while missing the SNARE motif [9].

The complexity of protein complements can be increased by either gene amplification, as in the just reported case of VAMP7 in plants, or by more sophisticated post-transcriptional regulation, as in the case of the human gene *SYBL1* encoding VAMP7. This gene is localized on the human-specific Xq/Yq pseudoautosomal region and, unexpectedly, it shows mono-allelic expression in both XY males and XX females because of X and Y inactivation [25]. The transcription silencing of the inactive alleles is achieved through epigenetic mechanisms [26]. Such a peculiar dosage compensation of *SYBL1* seems to be fundamental for human development. Indeed, copy number variations of a genomic region including *SYBL1* have been associated with genito-urinary birth defects [27], and with a form of intellectual disability affecting language skills [28].

The *SYBL1* gene is composed of eight exons, with exons 2–4 coding for the LD, and exons 5–8 coding for the SNARE motif and the TMD. Notably, *SYBL1* mRNA undergoes an extensive process of alternative splicing: transcripts are tissue- and stage-specifically expressed and were found in all cell lines tested [29]. They result in the production of VAMP7 protein variants with varying domain architecture. In particular, it has been reported that: (i) in addition to the main isoform VAMP7a, “minor” isoforms are expressed at physiologically relevant levels (40–60% of total VAMP7 mRNA), (ii) the existence of the corresponding protein variants is experimentally confirmed by immunological evidence and—of particular relevance to this study performed here—(iii) the isoform level is modulated in the brain from fetal to adult neurodevelopmental stages [29]. Apart from the largely investigated main isoform, which retains all exons and hence a complete domain architecture, only VAMP7b and VAMP7c variants have been (partially) characterized so far. Therefore, further minor variants should be investigated because these proteins show different domain architectures and LD-SNARE-TMD separation, which are likely to modulate interactions underlying functional specialization. This is just the case of the non-SNARE VAMP7b variant, which seems to play a special role in autophagosome–lysosome fusion [30] and results from the skipping of exon 6 and the downstream frameshift of the coding sequence [29]. Another non-SNARE variant is VAMP7i, which roughly corresponds to the LD alone [29]. However, non-LD VAMP7 variants have been found as well, namely VAMP7c and VAMP7dh. VAMP7c lacks 40 amino acids of the N-terminal region, which is subsequently unlikely to fold as a canonical LD. This variant does not retain the ability of VAMP7a to interact with the δ subunit of the AP-3 complex and hence it shows a different SCL [15]. Similarly, VAMP7dh localizes differently from the other variants as it is like a VAMP7a devoid of the LD [29].

In neuronal cells, effects on neuritogenesis of VAMP7i and VAMP7dh should be equal—or somewhat similar—to those observed with two artificial fragments of VAMP7; specifically, a fragment corresponding to the LD alone (like VAMP7i) was found to inhibit neuritogenesis, while the VAMP7_∆LD fragment (like VAMP7dh) promotes membrane fusion and neurite elongation [31].

This study aims to characterize splice variant VAMP7j, which results from in-frame skipping of exons 5 and 6. Among minor isoforms identified so far, VAMP7j is the most similar to the main isoform, as it retains all domains but the N-terminal half of the SNARE motif. Focusing on this variant was also prompted by evidence that VAMP7j is the most expressed minor isoform in the human brain, both in fetal and adult neurodevelopmental stages [29]. To shed light on the neuronal role of VAMP7j, we performed in silico and in vitro analyses, using recombinant isoforms and the SH-SY5Y cell line, a well-established human cellular model, overcoming the lack of isoform-specific antibodies. Evidence presented in this work suggests VAMP7j is an important player in the regulation of neurite growth and elongation pathway and highlights its engagement in the transport of L1CAM to the PM.

## 2. Results

### 2.1. VAMP7j Is a Non-SNARE Splice Variant and Regulates Neuritogenesis in Human SH-SY5Y Cells

**Figure 1:**
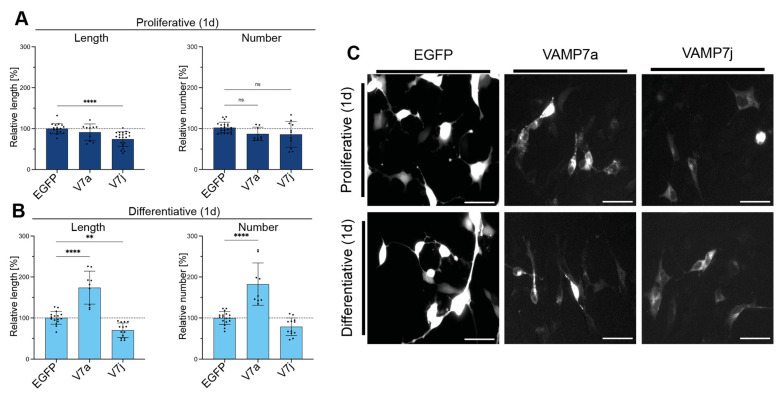
Overexpression of VAMP7a and VAMP7j isoforms in neuritogenesis assays on SH-SY5Y cells transfected with VAMP7a (V7a), VAMP7j (V7j), or EGFP control (expressed by the same plasmid vector). (**A**,**B**) Neuritogenic properties of the two splice variants are compared to the basic level of the EGFP control, in terms of neurite length (**left** side graphs) and neurite number (**right** side graphs). Only neurites longer than 50 µm are considered (see Section 4). Each point represents the aggregate value of at least 250 cells recorded in 10 different microscopy fields. All data represent the mean ± SD of at least three independent experiments. Significance at *p* ≥ 0.05 (ns), *p* < 0.01 (**), *p* < 0.0001 (****) is reported and has been determined by one-way ANOVA with Tukey’s correction. (**C**) Representative fields of cells transfected with VAMP7 isoforms. Scale bar: 50 µm.

VAMP7a, the main VAMP7 isoform, is known to induce neurite outgrowth in cell models [32], whereas the effect of VAMP7j on this process is still unknown. Therefore, we compared the effects of the two human VAMP7 isoforms in neurite outgrowth assays on transfected neuroblastoma-derived SH-SY5Y human cells. Specifically, cells were transfected with either one out of such two VAMP7 splice variants tagged with EGFP or with EGFP alone (same vector, as a control), in the presence or absence of the differentiating agent all-trans retinoic acid (RA). The neuritogenic properties of each isoform were then normalized to that of EGFP-transfected cells in terms of an increase in neurite length and number and then compared, as described in the Methods section. Twenty-four hours after transfection, VAMP7a was found to promote neuritogenesis, while having no meaningful effect on undifferentiated cells. Instead, VAMP7j reduced neurite elongation in both proliferative and differentiative conditions (Figure 1A–C). It is likely that this functional shift depends on the unique difference between the two proteins: VAMP7a has a complete and functional SNARE motif, whereas VAMP7j retains only the C-terminal hydrophobic heptads of the SNARE (hereinafter referred to as SNARE_C-ter_) and lacks its N-terminal half (SNARE_N-ter_).

The closed conformation is mediated in VAMP7a by intramolecular binding of SNARE_N-ter_ to the α1-β3 sub-region of the LD [13]. Since in VAMP7j, the absence of SNARE_N-ter_ puts the LD close to SNARE_C-ter_, we wondered if the SNARE_C-ter_ might somehow mimic the SNARE_N-ter_ in folding back to the LD in VAMP7j. Therefore, we generated a model for the cytoplasmic region of VAMP7j (residues 1–138, corresponding to LD + SNARE_C-ter_, without TMD and vesicular tail) in the closed conformation, using the VAMP7a crystal structure as a template (PDB ID: 4B93, including the LD and the SNARE_N-ter_). This model was used as a starting point for three 1 µs molecular dynamics (MD) simulations and compared with MD simulations of the crystal structure of the closed conformation of VAMP7a. Supplementary Figure S1 shows the Cα-RMSD (root mean square deviation of the protein backbone) at each time point of the simulation for the initial structure. This index quantifies how much a protein structure in a given time frame differs from the initial one. Moreover, the root mean square fluctuation (RMSF) index indicates how much a residue oscillates around its average position during a simulation. Residues with high RMSF values (>0.4 nm) are endowed with high conformational freedom and can therefore move more freely during the simulation. In simulations with VAMP7a, RMSD values quickly reach a plateau at 0.4 nm, indicating that the equilibrium structure is very similar to the initial one. The graphs confirm the high stability of the LD and show higher mobility for the SNARE motif; however, the mean average distance matrix, which plots the average distance of every residue from every other, indicates the SNARE motif remains bound to the LD. This agreement with previous experimental evidence [13] validates these MD simulations with VAMP7a as a reference for inferring the putative conformation of VAMP7j from the corresponding simulations. Indeed, when repeating the analyses for the closed conformation of VAMP7j, the results are quite different from those of VAMP7a, as simulations quickly reach RMSD values >1 nm, indicating that the closed conformation is unstable in VAMP7j. The RMSF and average distance plots further suggest this instability, as SNARE residues have high (>0.8 nm) RMSF values and a high average distance from the LD. Overall, these data indicate the newly positioned SNARE_C-ter_ of VAMP7j is unable to replace the missing SNARE_N-ter_ in intramolecular binding to the LD and therefore VAMP7j is unlikely to exist in closed conformation.

**Figure 2:**
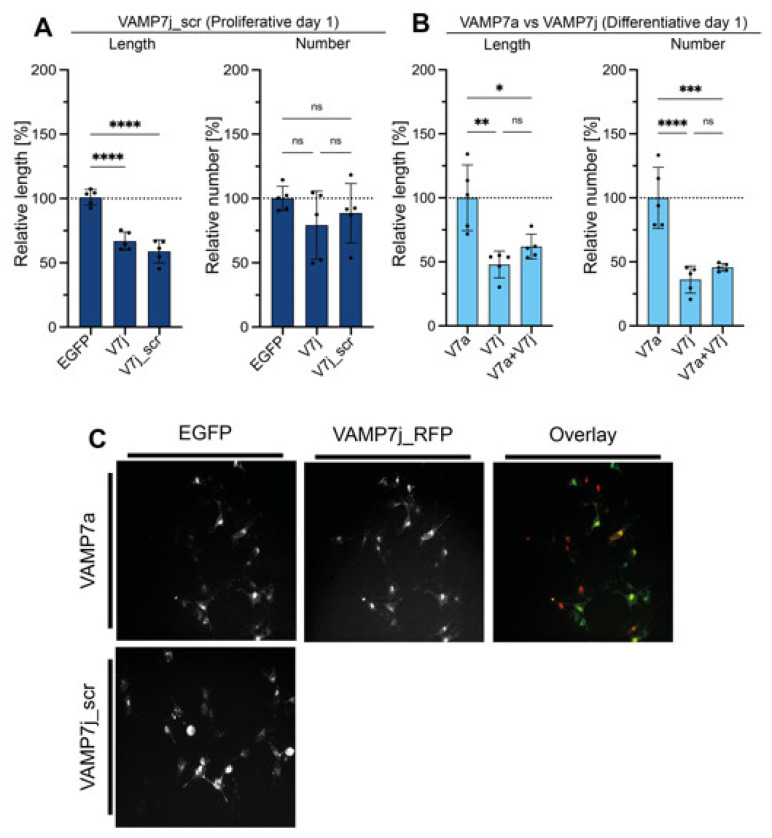
VAMP7j affects VAMP7a-mediated neurite growth independently on its SNARE motif. Neuritogenesis assays of (**A**) VAMP7j (V7j) compared to VAMP7j_scrambled (V7j_scr) in proliferative conditions, (**B**) VAMP7a (V7a) co-transfected with VAMP7j in differentiative conditions. All data represent the mean ± SD of at least five independent experiments. Significance at *p* ≥ 0.05 (ns), *p* < 0.05 (*), *p* < 0.01 (**), *p* < 0.001 (***), *p* < 0.0001 (****) is reported and has been determined by one-way ANOVA with Tukey’s correction. (**C**) Representative microscopy fields of conditions analyzed in panels (**A**,**B**). Scale bar: 50 µm.

However, we aimed to further investigate the role, if any, played by SNARE_C-ter_ in the ability of VAMP7j to inhibit neuritogenesis, due to the involvement of this region in membrane fusion. Indeed, the formation of the *trans*-SNARE complex takes place in two separate steps, from the membrane distal regions towards the proximal ones [17]. Priming and docking are mediated by the slow initial binding of the SNARE_N-ter_ of the VAMP SNARE motif to its SNARE partners on the target membrane, while subsequent binding of the corresponding halves of the SNARE_C-ter_ releases the energy needed to bring membranes together and drive their fusion [33,34,35,36]. Therefore, we designed a VAMP7j mutant with scrambled SNARE_C-ter_ sequence, i.e., where the heptadic frame of hydrophobic residues (which is crucial to the SNARE function) is disrupted. As shown in Figure 2, the effects on neuritogenesis of this mutant and wild-type (WT) VAMP7j are not meaningfully different, suggesting that (i) the remaining SNARE_C-ter_ of VAMP7j is as (un)functional as a scrambled half SNARE motif and (ii) VAMP7j-mediated inhibition of neurite outgrowth depends only on the absence of SNARE_N-ter_.

The loss of both intramolecular LD-SNARE binding and intermolecular SNARE-SNARE binding is therefore predicted to keep VAMP7j in open conformation. This in turn could influence VAMP7a domain interactions in membrane compartments, when considering the two isoforms sharing the same TMD and cytoplasmic tail. Finally, to test whether VAMP7j can modulate VAMP7a function, experiments were performed under conditions in which the main isoform exhibited the highest neuritogenic activity. Therefore, VAMP7j and VAMP7a were co-transfected in differentiating cells. Co-transfection with VAMP7 isoforms was set up previously [29] and has been confirmed over the years, allowing us to exclude the occurrence of unbalanced transfection efficiency with individual variants. As shown in Figure 2, we found VAMP7j to have a dominant negative effect, further suggesting it might act as a modulator of VAMP7a-mediated outcomes on neuritogenesis.

### 2.2. VAMP7a and VAMP7j Show Different Subcellular Localization Profiles

**Figure 3:**
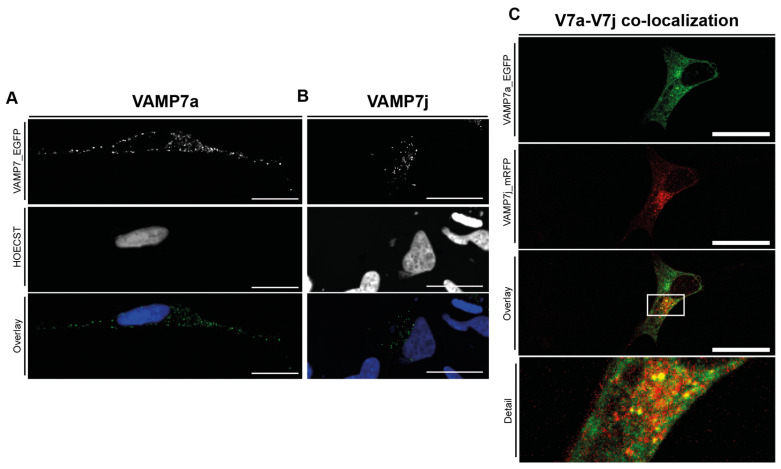
Localization of VAMP7-coated vesicles in SH-SY5Y cells. (**A**) VAMP7a vesicles (green, in the overlay image, where nuclei are blue) appear dispersed around the whole cell and its processes. (**B**) VAMP7j (green in the overlay image, where nuclei are blue) accumulates in the cell soma. (**C**) VAMP7a (green) and VAMP7j (red) co-localization (yellow) in co-transfection experiments. Scale bar: 25 µm. Detail image at the bottom corresponds to the magnification of the white square in the Overlay image.

Localization in one or more specific cell compartment(s) is a prerequisite for inferring the molecule function(s) within cell physiology. Indeed, in our previous studies, it was reported that human VAMP7 isoforms show different SCL profiles, only partially overlapping with that of the main isoform. Since these studies were performed in non-neuronal HeLa cells [29], we decided to explore the distribution of VAMP7-coated vesicles in neuroblastoma-derived SH-SY5Y cells as a proxy of their neuronal function. As shown in Figure 3A, VAMP7a-coated vesicles are widespread throughout the cell soma and growing neurites, without accumulating at any specific point. In contrast, VAMP7j-coated vesicles show peculiar distributions (Figure 3B) and accumulate in the cell soma.

To shed more light on the trafficking routes of the two isoforms, we performed co-transfection experiments. Mander’s coefficient for VAMP7a co-localization with VAMP7j is 0.24, and it increases to 0.53 for VAMP7j co-localization with VAMP7a, indicating only a small fraction of VAMP7a co-localizes with VAMP7j, whereas roughly 50% of VAMP7j co-localizes with VAMP7a. These two isoforms only partially co-localize within vesicles, and double-positive vesicles were enriched in the cell soma (Figure 3C). These data are compatible with the existence of three vesicle sub-types, positive for (i) VAMP7j, (ii) VAMP7a, and (iii) both variants. Moreover, VAMP7j could be dominant over VAMP7a in defining its SCL. These hypotheses will need further functional evidence for final confirmation; however, they agree with previous observations indicating the longin domain is crucial in determining VAMP7 subcellular localization [15].

### 2.3. VAMP7j Determines L1CAM Subcellular Localization in Human SH-SY5Y Cells

Once the functional dominance of VAMP7j over the main isoform VAMP7a is established, we further investigated VAMP7j-mediated inhibition of neurite outgrowth. During rat development, VAMP7a has been shown to be crucial for the positioning of L1CAM at the plasma membrane, suggesting that this may contribute to its pro-neuritogenic activity [37]. However, such evidence of co-localization of VAMP7a with L1CAM was obtained in rat PC12 cells, i.e., in a non-human cellular system [8]. As reported in the introduction, VAMP7j is the most expressed minor isoform in both fetal and adult human brains and, crucial for the present study, its expression has also been validated in human SH-SY5Y cells [29].

**Figure 4:**
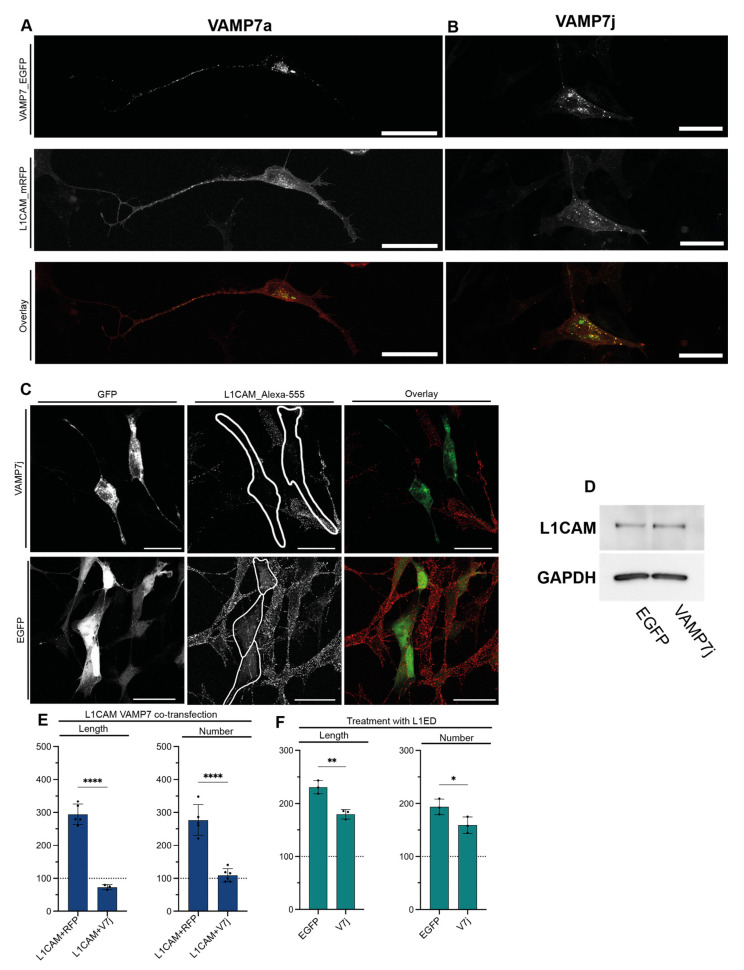
Effects of VAMP7j on L1CAM-mediated neuritogenesis. (**A**) VAMP7a and L1CAM do not co-localize in SH-SY5Y cells. (**B**) Co-localization is observed between VAMP7j and L1CAM in SH-SY5Y cells. (**C**) Maximum projections of transfected cells stained (without permeabilization) with L1CAM antibody. Cells transfected with VAMP7j do not show localization of L1CAM at the plasma membrane. (**A**–**C**) Scale bar: 25 µm. (**D**) Expression of VAMP7j does not alter the overall L1CAM protein expression. (**E**,**F**) Neuritogenic properties of (**E**) cells co-transfected with L1CAM and VAMP7j or (**F**) transfected with VAMP7j and treated with 1 nM L1CAM ectodomain (L1ED). VAMP7j can reduce L1CAM ectodomain effect. Significance at *p* < 0.05 (*), *p* < 0.01 (**), *p* < 0.0001 (****), is reported as determined by one-way ANOVA with Tukey’s correction.

Therefore, we performed co-transfection experiments in human SH-SY5Y cells by using L1CAM with VAMP7a or VAMP7j. Notably, co-transfection of VAMP7a and L1CAM resulted in only poor co-localization of the intracellular pool of the two proteins, as Pearson’s and Mander’s coefficients for VAMP7a co-localization with L1CAM are 0.2 and 0.4, respectively (Figure 4A). Instead, VAMP7j showed co-localization with the intracellular pool of L1CAM in human SH-SY5Y (Pearson’s and Mander’s coefficients for VAMP7j co-localization with L1CAM are 0.65 and 0.81, respectively), and a particular enrichment of co-localization spots in the cell soma (Figure 4B).

To assess membrane transport of L1CAM in human SH-SY5Y cells, we stained the membrane pool of endogenous L1CAM in both VAMP7j-transfected and control cells (labeled without permeabilization). As shown in Figure 4C, L1CAM is not found at the PM in VAMP7j-transfected cells, and this does not depend on impaired expression, as the total amount of L1CAM does not show any significant change in Western blot experiments (Figure 4D). To account for any possible effect of transfection on the L1CAM positioning, we assessed the presence of L1CAM at the PM in EGFP-transfected cells. Notably, we did not find any difference between transfected and control cells, indicating the absence of L1CAM from the PM is directly dependent on VAMP7j co-expression.

Given the relevance of VAMP7j in determining the subcellular sorting of L1CAM in human cells, we assessed its ability to influence L1CAM-mediated neuritogenesis. VAMP7j exhibited a dominant negative effect even on L1CAM, fully repressing its functionality in co-transfection experiments (Figure 4E). L1CAM signaling can be triggered by the treatment of cells with the L1CAM extracellular domain (L1CAM_ED), which is a potent inducer of neuritogenesis, even at concentrations as low as 10 pM in SH-SY5Y cells [7]. VAMP7j transfection can only partially reduce L1CAM_ED effects (Figure 4F). However, this is not unexpected since L1CAM_ED, in addition to preferential homophilic binding to L1CAM, is also capable of heterophilic binding with other neuronal CAMs [38,39,40].

Finally, we could exclude that the observed difference in L1CAM co-localization (with VAMP7j in human SH-SY5Y and with VAMP7a in rat PC12) depends on different experimental conditions because, by repeating the co-transfection experiments with PC12 cells, previously published evidence was confirmed (see [8] for details).

### 2.4. VAMP7j Is a Human Isoform, Shared with Primates and Absent in Neural Rat Tissues and Cells

The hypothesis that VAMP7j might be a peculiar feature of SH-SY5Y cells rather than an important player in human neurodevelopment can be excluded based on several lines of evidence. Indeed, it has been reported [29] that (i) this isoform is present in all tested human cell lines, tissues, and organs, (ii) among minor isoforms of VAMP7, it is the most expressed in human tissues and (iii) VAMP7j shows stage-specific expression in the human brain. Since species-specific variation in the splicing profile is widely documented for many mammalian genes, species-specific splicing events leading to VAMP7j production may underlie the difference between human and rat cells in the regulation of neuronal L1CAM trafficking. Therefore, we investigated the presence or the absence of the specific isoform VAMP7j in rat tissues, using amplification of the shared isoform VAMP7a as a positive control.

**Figure 5:**
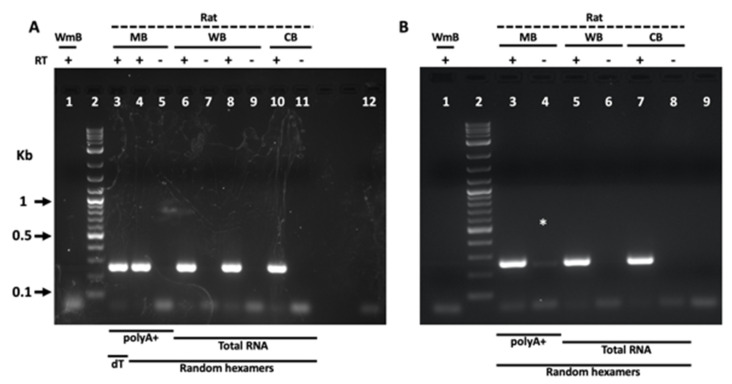
VAMP7j is not expressed in the rat brain. Representative images of the agarose gel (2%) electrophoresis showing the end-point RT-PCR products for rat VAMP7, spanning the coding region from exon 4 to exon 7. (**A**) PCR amplicon obtained by combining rat rVAMP7_ex4F_2 (localized on exon 4) with rVAMP7_ex7R_2 primers (localized on exon 7) and the following templates: polyA+ RNA from the midbrain (MB, lanes 3–5) and total RNAs from the whole brain (WB, lanes 6–9) or from the cerebellum (CB, lanes 10 and 11) of rats, retrotranscribed with (RT+, lanes 3, 4, 6, 8, 10) or without (RT−, lanes 5, 7, 9, 11) the reverse transcriptase enzyme. The same polyA+ RNA was retrotranscribed with oligo-dT (lane 3) or random hexamers (lanes 4 and 5). (**B**) Nested RT-PCR results of the same RNA samples as in panel (**A**), pre-amplified with rVAMP7_ex2F (localized on exon 2, thus upstream to exon 4) and rVAMP7_ex8R (localized on exon 8, thus downstream to exon 7) primers and then used as PCR templates with rVAMP7_ex4F_2 and rVAMP7_ex7R_2 (nested) primers (i.e., the same primers as in the PCR of the panel (**A**)). (*) Spill-over of the sample from the adjacent well #3 during loading. The cDNA obtained from total RNAs extracted from the whole mouse brain (WmB, lane 1 panels (**A**,**B**)) and no cDNA template (lane 12, panel (**A**) and lane 9 panel (**B**)) were used as negative controls for the PCRs. By combining rat VAMP7_ex4F_2 with VAMP7_ex7R_2 primers, a doublet of 263 bp and 104 bp is theoretically expected, corresponding to VAMP7a (from exon 4 to exon 7) and VAMP7j (exons 4 and 7) isoforms, respectively. In both panels, all lanes with rat RT+ show only the amplicon for the VAMP7a isoform (263 bp), representing the loading control. No differences are noticeable between oligo-dT and random hexamers used in the first-strand step of the cDNA synthesis (lanes 3 and 4, panel (**A**)). DNA size marker of 1 Kb plus ladder (New England Biolabs, Ipswich, MA, USA) was loaded in lane 2 (both panels).

We first amplified the coding region in between exons 4 and 7, revealing that only the VAMP7a isoform is expressed, both in the whole brain and in specific brain sub-regions of rats (Figure 5A). Then, to increase the amplification rate of low-expression transcripts, a nested-PCR approach was exploited. Nonetheless, the VAMP7j isoform was still undetectable in postnatal neural tissues of rats (Figure 5B), contrary to its reported presence in human adult tissues [29].

Lastly, a VAMP7j-specific primer (spanning the junction of exons 4 and 7 of the rat sequence) was used in qPCR experiments, and once again the expected amplicon was not found in the rat brain. This suggests that the skipping of exons 5 and 6 is unlikely to be evolutionarily preserved in the rat brain. Subsequently, VAMP7-mediated trafficking of L1CAM in rats and humans could be handled through separate mechanisms. To gain further insights into the species-specific profile of the splicing event leading to expression of VAMP7j, we performed a genome- and transcriptome-wide analysis in silico. A preliminary inspection of GenBank/EMBL entries (performed on 20 June 2023) showed that both the 8-exon structure and the X chromosome location of human *SYBL1* are shared with mammalian orthologues, whereas *SYBL1* genes from other vertebrates (e.g., chicken and zebrafish) have an autosomal location and a varying exon structure. Since the conserved 8-exon structure of mammalian *SYBL1* genes might allow (at least theoretically) other mammals to share VAMP7j with humans, publicly available transcriptomic data were investigated. Experimental datasets available at the NCBI BLAST portal were screened (i.e., excluding “predicted” sequences), searching for the exon 4–7 junction in representative species, and some positive hits were found in the Sequence Read Archive (SRA). Data shown in Supplementary Table S1 confirm no expression of VAMP7j in either rat or mouse, while a few matches from *O. cuniculus* only concern non-neural tissues (kidney, heart, and skeletal muscle). Most intriguingly, VAMP7j mRNAs are found in several human tissues (according to previously published experimental evidence) and humans share the expression of this isoform in the CNS only with primates.

### 2.5. VAMP7j Phosphorylation Affects L1CAM-Dependent Neurite Growth

VAMP7a activity is regulated by phosphorylation of tyrosine (Y) 45 of the LD. When members of the cSrc family kinases phosphorylate VAMP7a_Y45, membrane fusion is favored. Instead, when Y45 is dephosphorylated, the LD folds back onto the SNARE motif, thus preventing its participation in the fusion complex [14]. However, Y45 is unlikely to play the same role in VAMP7j, i.e., to disturb the intramolecular LD-SNARE binding that leads to the closed conformation, because the LD partner (SNARE_N-ter_) is absent in VAMP7j.

Comparative MD simulations illustrated above also suggest VAMP7j closed conformation is unstable, hence advising that the phosphorylation of Y45, if any, would play another role in VAMP7j functions. However, to assess whether the conformation of VAMP7j could eventually be modulated by the phosphorylation of Y45, we used the CHARMM-GUI tool to insert the Y45p modification in both the VAMP7a structure and VAMP7j model generated for MD simulations (Supplementary Figure S1). Seemingly, Y45 plays a marginal role, coming in contact with the extreme C-terminal of the VAMP7j SNARE. Simulations shown in Supplementary Figure S2 agree with the experimental observation on VAMP7a [14] and confirm the predicted open conformation for both unphosphorylated and phosphorylated VAMP7j.

**Figure 6:**
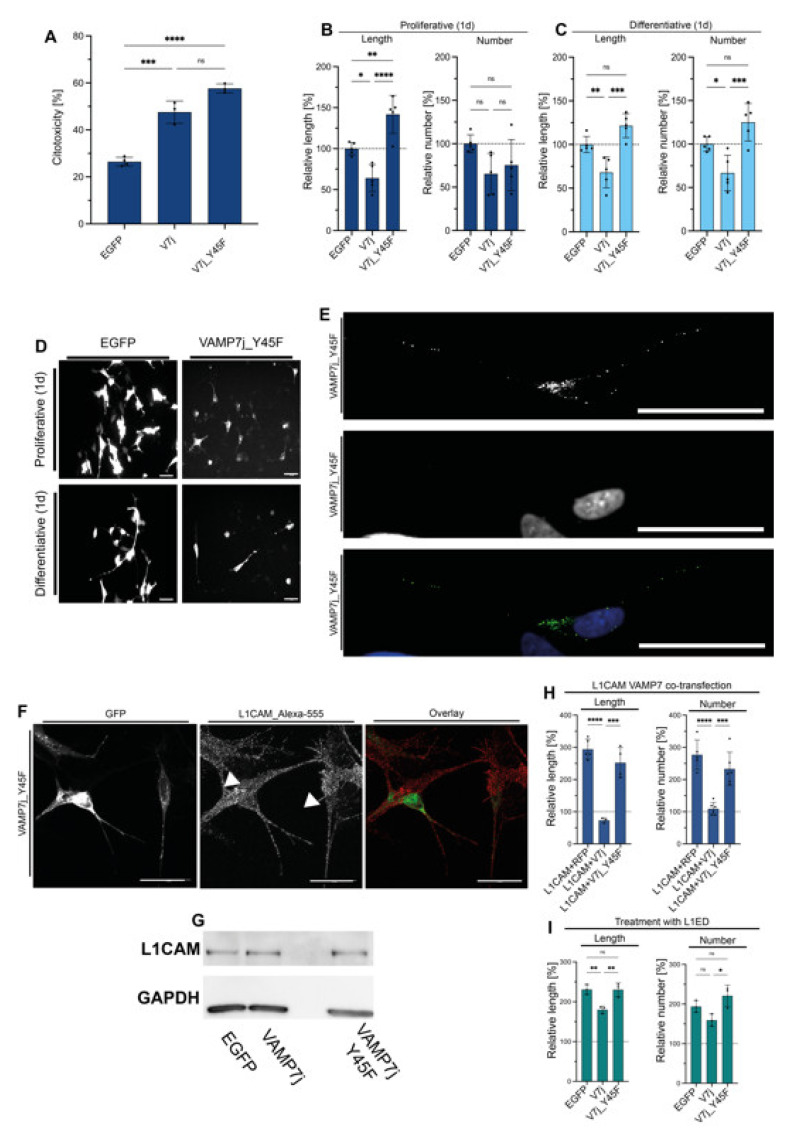
Effects of VAMP7j_Y45F on neuritogenesis. (**A**) Normalized cytotoxicity of the VAMP7j mutant. (**B**) Neuritogenic effect of VAMP7j_Y45F vs. EGFP control and VAMP7j in proliferative conditions. All proteins affect neurite length and do not modify the average number of neurites. (**C**) Neuritogenic properties of VAMP7j_Y45F vs. EGFP control and VAMP7j in differentiative conditions. Unlike VAMP7j, VAMP7j_Y45F does not interfere with neurite outgrowth. (**D**) Representative microscopy fields of VAMP7j_Y45F-transfected cells in proliferative and differentiative conditions compared to EGFP-transfected cells. Scale bar: 50 µm. (**E**) Confocal microscopy detail of a cell transfected with VAMP7j_Y45F (green in the Overlay image). Vesicles are found in both the cell soma and neurites. Scale bar: 25 µm. (**F**) Maximum projections of transfected cells stained with L1CAM antibody (red in the Overlay image, where GFP is green). Cells transfected with VAMP7j_Y45F show localization of L1CAM at the PM (white arrows). (**G**) Expression of VAMP7j_Y45F do not alter L1CAM total expression levels. (**H**,**I**) Neuritogenic properties of cells transfected with VAMP7j and treated with 1 nM L1CAM ectodomain (L1ED). Significance at *p* ≥ 0.05 (ns), *p* < 0.05 (*), *p* < 0.01 (**), *p* < 0.001 (***), *p* < 0.0001 (****), is reported as determined by one-way ANOVA with Tukey’s correction.

To gain insights into a possible role of Y45 phosphorylation in VAMP7j, we designed the Y45F non-phosphorylatable variant of VAMP7j for tests in proliferating SH-SY5Y cells. VAMP7j_Y45F toxicity was comparable to that of WT VAMP7j, but this mutant was no longer able to repress neuritogenesis (Figure 6A–C). When the VAMP7j_Y45F-coated vesicles distribution was considered, we found they were widespread throughout the cell (Figure 6D). We also assessed the relevance of Y45 residue on VAMP7j co-localization with L1CAM. Both VAMP7j and its mutant version VAMP7j_Y45F co-localized with the intracellular pool of L1CAM in human SH-SY5Y (Pearson’s and Mander’s coefficients for VAMP7j co-localization with L1CAM are 0.65 and 0.81, and for the VAMP7j_Y45F co-localization with L1CAM are 0.53 and 0.67, respectively). Of note, co-localization was observed only in the soma of VAMP7j transfected cells and on both soma and neurites of VAMP7j_Y45F transfected cells.

After staining the membrane pool of endogenous L1CAM in VAMP7j-, VAMP7j_Y45F-transfected and control cells, the localization of L1CAM at the PM was observed in VAMP7j_Y45F-transfected cells, but not in VAMP7j-transfected ones (Figure 6E), even though the total amount of L1CAM did not show any significant change in Western blot experiments (Figure 6G).

Unlike VAMP7j, VAMP7j_Y45F was unable to antagonize L1CAM-overexpression-dependent neuritogenesis in co-transfection experiments (Figure 6H). Figure 6I shows that the partial reduction in L1CAM_ED effects can only be achieved through VAMP7j transfection. Therefore, the membrane transport of L1CAM in human SH-SY5Y cells seems to be further modulated by the phosphorylation of VAMP7j.

### 2.6. The Phosphorylation of VAMP7j Modulates Binding to LRRK1

**Figure 7:**
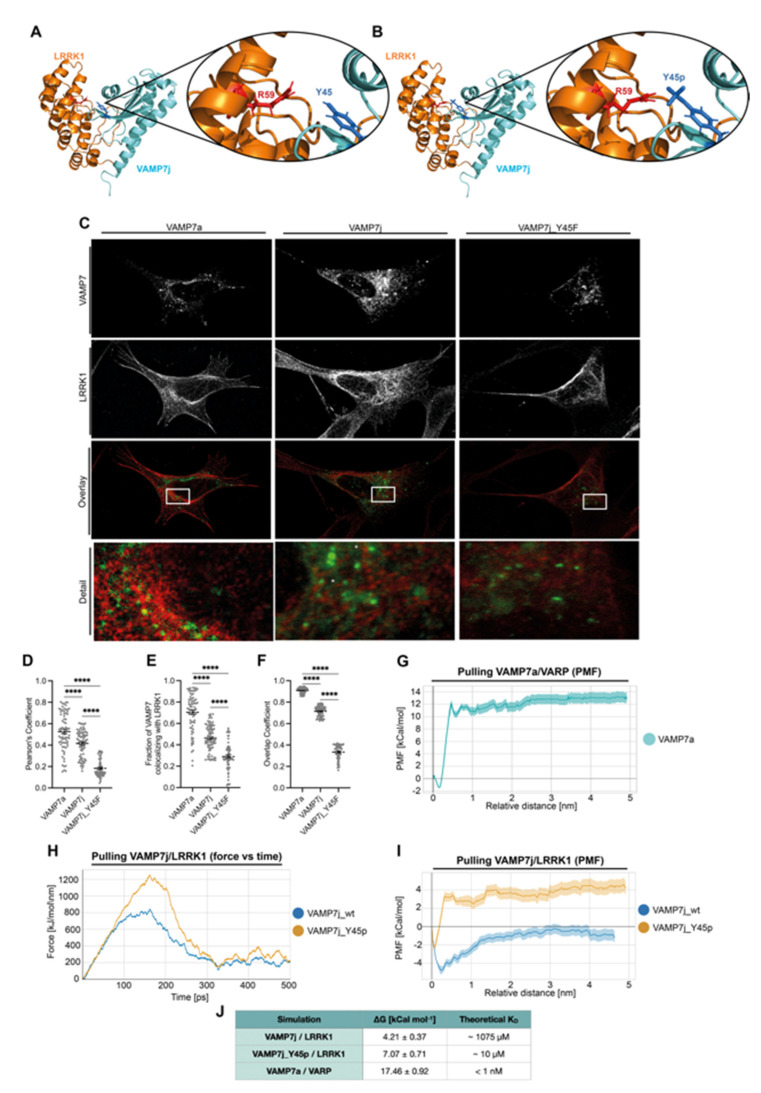
Binding to LRRK1 depends on VAMP7j Y45 phosphorylation. Docked models for the interaction between unphosphorylated (**A**) and phosphorylated (**B**) VAMP7j in complex with LRRK1. Addition of a phosphate group could strengthen the bond between LRRK1_R59 and VAMP7j_Y45. (**C**) Co-localization between VAMP7 (green) and LRRK1 (red). Bottom “Detail” images are the magnification of white squares. (**D**–**F**) Pearson’s, Mander’s and Overlap indexes determined with BIOP JaCoP. Error bars indicate SEM. Significance at *p* < 0.0001 (****), is reported and has been determined by one way ANOVA with Tukey’s correction. (**G**) Potential mean of force for the pulling simulation of the VAMP7a/VARP crystal structure. (**H**) Forces vs. time graph of the pulling simulation of VAMP7j from LRRK1. A higher force is required to dissociate the phosphorylated complex. (**I**) Free energy variation in the VAMP7j/LRRK1 dissociation process. (**J**) Estimated values for ∆G_bind_ and K_D_ derived from the simulations.

To provide a rationale for this latter finding and considering the role of VARP and LRRK1 in mediating the positioning of VAMP7a-coated vesicles [16], we designed models of ankyrin repeat regions for both proteins and used them for docking simulations against VAMP7j in open conformation. Models of VAMP7j were obtained as representative frames extracted from MD simulation. Docking simulations were performed through assigned attraction between VAMP7j and regions of VARP (residues 668–698) and of LRRK1 (86–116), which are known to bind VAMP7a [41]. The best docking pose for VARP on VAMP7j was found to be likely unstable (Supplementary Figure S3), missing all relevant contacts described in the VAMP7a-VARP crystal structure, according to evidence that VARP binds to VAMP7a LD in closed conformation, but not to the LD alone [17]. Conversely, the binding of LRRK1 seems to be more relevant. LRRK1 binds to the αp-β3-β4 sub-region of the LD in the proposed pose, which is known to mediate several protein–protein interactions of VAMP7 [11]. Moreover, the model shows a polar bond between LRRK1_R59 and VAMP7j_Y45, which could be strengthened by the negative charge provided by the phosphate group upon phosphorylation (Figure 7A,B).

When we assessed co-localization between LRRK1 and VAMP7a, VAMP7j or VAMP7j_Y45F, we found strong co-localization only for VAMP7a. Although staining of LRRK1 and VAMP7j showed some similarities, with some vesicles positive for VAMP7j surrounded by LRRK1 positive foci, it also showed regions of mutual exclusion. Such similarities were less marked in VAMP7j_Y45F transfection (Figure 7C). Pearson’s correlation, Mander’s and overlap coefficients (Figure 7D–F) indicate only a partial co-localization between LRRK1 and VAMP7j, which is strongly reduced in VAMP7j_Y45F transfection. Such a result is compatible with the LRRK1 mechanism in the context of dynein loading, as reported by Kedashiro and co-workers [42]. Moreover, it is coherent with the assumption that only a fraction of the VAMP7j pool is phosphorylated in the assayed conditions and, overall, it suggests a transient interaction between LRRK1 and VAMP7j.

The dissociation constant (K_D_) of the LRRK1::VAMP7 complexes was estimated by Umbrella Sampling, a type of MD simulation in which an external force is applied to pull apart two members of a complex along a reaction coordinate. This pulling simulation is used as a starting point to infer the free energy landscape of the dissociation. The ∆G_bind_ between the two species can be computed as the difference between the minimum and maximum free energy value and used to determine K_D_. This value is determined in ideal conditions and constitutes a rough estimate of an experimentally determined K_D_; however, it can still be used to compare the relative force of interactions between similar complexes.

Even though pulling of LRRK1 from VAMP7a would have been the best control for such simulations, we opted to simulate the dissociation of the VARP/VAMP7a complex, for which a solved crystal structure is available. Instead, the absence of structural information on the binding of LRRK1 to VAMP7a makes it challenging to generate a model. Specifically, it is neither known whether LRRK1 binds to VAMP7a in open or closed conformation, nor if and how the SNARE motif interacts with LRRK1.

Moreover, Schäfer and co-workers [17] used isothermal titration calorimetry to infer K_D_ for the interaction between VARP and VAMP7a, which could be used as a reference to validate our in silico data. Before starting the pulling simulation, energy was minimized, and the system was equilibrated to 310° K and 1 bar to simulate realistic experimental conditions.

In agreement with the already available data, pulling of the VARP-VAMP7a complex resulted in a high release of free energy (∆G_bind_ = 17.46 ± 0.92 kCal/mol), suggesting the complex is highly stable (Figure 7G,J). However, we estimate the K_D_ of this interaction to be in the sub-nanomolar range (i.e., more than 4 orders of magnitude lower than the experimentally determined one). This is not unexpected, since in solution VAMP7a can switch between the open and closed conformation, while our simulation is performed in ideal conditions where VAMP7 can only explore the closed conformation.

Simulations of the VAMP7j/LRRK1 pulling showed different force vs. time curves (Figure 7H) and despite the identical original orientation, a higher force is required to fully dissociate the VAMP7j_Y45p/LRRK1 complex. The potential mean of force was calculated using the WHAM method to assess the relative stability of the complexes (Figure 7I). Pulling of the VAMP7j_Y45p_LRRK1 complex results in a lower energy minimum than the unphosphorylated counterpart with K_D_ in the low- and mid-micromolar range, suggesting a higher stability of the phosphorylated complex. The absence in VAMP7j of SNARE_N-ter_ is likely to account for the observed change in the effect of Y45 phosphorylation. Indeed, such an absence is sufficient alone to impair formation of the closed conformation, and this in turn is able per se to prevent binding of VARP to VAMP7j. Therefore, Y45 phosphorylation can only influence residual interactions with the LD in open conformation, like the one with LRRK1.

## 3. Discussion

The three longin subfamilies are conserved in all eukaryotes, and even though VAMP7 is suggested to play a special role in neurite outgrowth, elongation, and neurodevelopment, only its main splice isoform VAMP7a has so far been extensively characterized. Indeed, evolutionary variation in alternative splicing profiles is well known to progressively increase [43] transcriptomic and proteomic complexity in higher eukaryotes. This applies in particular to the neural proteome and mammals, as even subtle adjustments at the molecular and cellular level may finally result, at the neural network level, in improved cognitive abilities and behavioral complexity [44,45,46,47,48,49].

Therefore, the characterization of alternative splice variants of proteins that, like VAMP7, are involved in neuronal differentiation and neurodevelopment, becomes of special interest, and this prompted us to focus on human VAMP7j. This isoform is interestingly characterized by two relevant features: among all the “minor” splice variants of human VAMP7 identified so far, it is the most expressed (after the main isoform VAMP7a) in the human brain and its expression is modulated in the transition from the fetal to adult stage [29]. Furthermore, VAMP7j shares almost the entire domain architecture with VAMP7a, except for the lack of the N-terminal half of the SNARE motif.

In this work, we overexpressed VAMP7a and VAMP7j in SH-SY5Y cells, which represent an established model system to study human neuronal differentiation and a cell line where the expression of all human VAMP7 isoforms has been already documented [29]. Indeed, overexpression under the control of the constitutive, strong, CMV promoter allowed us to greatly increase the expression of both splice variants, without considering the background level of the other ones.

The neurite outgrowth assays reported in this work indicate that VAMP7j is capable of exerting a dominant negative effect over VAMP7a- and L1CAM-mediated neurite sprouting and elongation. This effect seems to be independent of membrane fusion and more likely depends on interactions of the longin domain.

Indeed, the absence in VAMP7j of the N-terminal half of the SNARE motif no longer allows for the intramolecular LD-SNARE binding leading to the closed conformation, as suggested by MD simulations. Evidence that the effect of VAMP7j on neuritogenesis is unaffected by scrambling the heptadic register of the residual C-terminal half of the SNARE motif further supports the non-SNARE functionality of this longin isoform.

A functional reshaping of the domain architecture with the loss of the SNARE motif in longins is known to be mediated also by gene amplification and subsequent divergence of paralogues, as observed with animal Sec22a/c and plant phytolongins (see Section 1 for more details). Therefore, it is not surprising to find that such functional reshuffling is also achieved through additional molecular mechanisms such as alternative splicing.

To gain insights into the involvement of VAMP7j in cellular pathways, we investigated its SCL at the level of vesicle pools, and the regulation by phosphorylation of Y45, an LD-embedded residue. VAMP7j-coated vesicles are found in the perinuclear compartment, with localization dependent on Y45 phosphorylation. Indeed, upregulation of the non-phosphorylatable mutant VAMP7j_Y45F resulted in the migration of VAMP7j-coated vesicles from the perinuclear region to the cell periphery and in the loss of the repressive abilities and dominance of VAMP7j.

Docking and MD simulations suggest VAMP7j does not interact with VARP, a cognate partner of VAMP7a, whereas it preferentially binds LRRK1 upon phosphorylation. VAMP7j binding to LRRK1 might link vesicles to dynein and favor their retrograde transport and accumulation in the perinuclear compartment. This mechanism is particularly relevant to the transport of L1CAM, as the ectopic expression of WT VAMP7j (but not that of VAMP7j_Y45F) results in the loss of L1CAM at the PM, as suggested by both L1CAM membrane staining and the reduced effect of a L1CAM_ED treatment.

Previous studies in rat PC12 cells reported that trafficking of L1CAM, a pivotal player in mammalian and human neurodevelopment, depends on VAMP7a [8]. However, while confirming this interaction in the same rat cells, we did not find any co-localization in the intracellular pool of L1CAM and VAMP7a in the human SH-SY5Y cell line, because in these cells L1CAM trafficking is regulated with VAMP7j rather than with VAMP7a. Moreover, we neither found VAMP7j expression in rat PC12 cells nor in rat brain tissues, hinting at a different, and possibly more finely tuned, mechanism in humans for the regulation of L1CAM transport. L1CAM plays a central role in neurodevelopment, as a molecular cue sensed by the axonal growth cone to establish proper connections in the forming neural network. Even though the in silico evidence shown in Supplementary Table S1 is of course preliminary, three points have to be considered: (i) wide expression of VAMP7j in human tissues as inferred in silico is in agreement with the experimental evidence [29]; (ii) the absence of positive hits from the Rodentia Sequence Reads Archive is also in agreement with the experimental evidence on rats (this work); (iii) publicly available transcript datasets are partial for some species, but the aggregate transcriptomic data from wider taxonomic groups are large enough to significantly reduce the risk of false negatives. This suggests that CNS expression of VAMP7j, restricted to humans and other primates, is meaningful and indeed it agrees with the experimental evidence on VAMP7j as the most expressed and modulated minor VAMP7 isoform in the brain [29]. Such an evolutionary sophistication in the splicing profile of VAMP7 adds a further level in the regulation of L1CAM transport in neural cells (evidence from this work). Therefore, it is tempting to speculate this might be functional in turn to a more finely tunable control of the neural network formation in the CNS of humans and primates, possibly associated to their higher cognitive capacities.

In the field of research into “what makes us humans”, the 2006 discovery of Human Accelerated Regions (HARs) [50] has sparked great interest into the accelerated evolution of non-coding sequences conserved in humans. HARs are likely to function as enhancers of gene regulation and show significant enrichment in neurodevelopment, perhaps underlying, at least in part, the higher cognitive functions of humans [51,52]. Together with the emerging role of lncRNAs as drivers of human brain evolution [53], human brain-specific regulation has also been reported for spatial gene expression [54], chromatin methylation [55], and expressed transposable elements [56]. In addition, the human brain has long been known to display the most complex pattern of alternative splicing, thereby producing diverse protein isoforms compared to other tissues, and this has been associated with a more sophisticate neurodevelopmental program [57]. In this context, human and/or primate-specific profiles of *SYBL1* alternative splicing may lead to the production of peculiar VAMP7 variants with special neurodevelopmental functions. Indeed, the characterization of the ability of VAMP7j to regulate the trafficking of L1CAM, a regulator of neuritogenesis, adds a further element of knowledge to the complex network of new or improved mechanisms underlying the peculiar features of human neurodevelopment and cognitive functions.

## 4. Materials and Methods

### 4.1. Cell Culture and Transfection

Human neuroblastoma cell line SH-SY5Y was cultured as reported [58,59] using Dulbecco’s Modified Eagle Medium/Nutrient Mixture F-12 with GlutaMAX™ supplement (DMEM/F-12; Invitrogen Life Technologies, San Giuliano Milanese, Italy), supplemented with 10% heat-inactivated fetal bovine serum (FBS; Euroclone, Pero, Italy) and 25 μg/mL of gentamicin (Sigma Aldrich, Milano, Italy) (growth medium), in a humidified atmosphere of 5% of CO_2_ in air at 37 °C. In transfection experiments, cells were seeded in a 24-well plate (25,000 cells/well) coated with a gelatin (Sigma Aldrich, Milano, Italy)/poly-L-lysine (Invitrogen, San Giuliano Milanese, Italy) solution. In experiments with differentiated cells, 24 h after cell seeding, the growth medium was replaced by differentiating medium (DMEM/F12, 2% FBS, 1 µg/mL all-trans retinoic acid (RA) and 25 µg/mL gentamicin. PC12 cells were cultured in RPMI supplemented with 15% FBS and 25 μg/mL gentamicin. Cells were transfected using lipofectamine 2000 (Invitrogen, San Giuliano Milanese, Italy) optimizing the manufacturer’s protocol. In particular, 2.5 × 10^4^ cells were transfected with 0.35 µg DNA and 0.5 µL lipofectamine, using the plasmid expressing EGFP alone as the control. Transfection medium was replaced by the culture medium 6 h after transfection. In experiments combining transfections and differentiation, differentiation medium was added 6 h after transfection and cells were observed 24 h after transfection.

### 4.2. VAMP7 and L1CAM Clones

Human VAMP7a and VAMP7j coding sequences (CDS) were cloned in either pEGFP_N1 or pmRFP_N1 vectors, as already described [29]. Human L1CAM CDS was cloned in either a modified version of pEGFP_N1 or pmRFP_N1, as described [6,7]. VAMP7j_Y45F mutant was produced using QuickChange site-directed mutagenesis kit (Agilent Technologies, Cernusco sul Naviglio, Italy) following the manufacturer’s protocol.

The primer pair was designed as follows: VAMP7j_Y45F_Fwd: 5′-CCTTCTGAAAATAACAAACTAACGTTCTCACATGGCAATTATTTG-3′ and VAMP7j_Y45F_Rv: 5′-CAAATAATTGCCATGTGAGAACGTTAGTTTGTTATTTTCAGAAGG-3′. VAMP7j_scrambled mutant was synthesized as a synthetic DNA fragment (GeneArt service; Life Technologies) so that SNARE_C-ter_ would be scrambled to: TLSASTFKVTRN.

### 4.3. Neuritogenesis Assay

Neurite outgrowth was measured as reported [6,7]. Briefly, transfected cells were counted, and neurite length was measured by tracing the trajectory of each neurite from its tip to the junction between the neurite and cell body. Only neurites longer than 50 µm were considered [60]. The neuritogenic properties were analyzed in terms of total neurite length/no. of cells (aggregate length of all cellular processes divided by cell number) and no. of neurites/no. of cells. To account for basal neurite growth, values were then normalized to the untreated proliferative control and reported as a percentage. Each experiment was performed in at least three independent replicates, and each point represents the aggregate value of at least 250 cells, recorded in 10 representative fields of each well.

### 4.4. Cell Fixation and Immunofluorescence

To check the SCL of transfected proteins, cells were fixed in 4% paraformaldehyde (PFA) for 15 min on ice and, if needed, incubated with 10 µg/mL Hoechst 33258 (Invitrogen) in PBS for 20 min. Finally, coverslips were mounted with a Mowiol mounting medium. After 24 h of polymerization, samples were observed using a Leica SP5 confocal microscope. For immunofluorescence experiments, fixed samples were permeabilized with ice-cold 100% methanol for 10 s and blocked in 0.5% BSA in PBS for 45 min at room temperature (RT). Incubation with the primary antibody (Table 1; providers details are the followings: ThermoFisher, Rodano, Italy, ABCAM, Cambridge, UK, Abnova-Prodotti Gianni, Milan, Italy) was performed for 90 min at RT and the antibody was diluted in 3% BSA in PBS.

The secondary antibody (Alexa Fluor 544; ThermoFisher) was diluted in 0.5% BSA and incubated for 45 min at RT. Finally, coverslips were mounted with Mowiol mounting medium. After 24 h of polymerization, samples were observed using a Leica SP5 confocal microscope. In L1CAM membrane-staining experiments, cells were fixed for 15 min in PFA without permeabilization with methanol. Co-localization was determined via the Pearson’s, Mander’s and Overlap coefficients computed by ImageJ v1.54.

### 4.5. RT-PCR

PolyA+ or total RNAs (kind gifts of Dr. Dario Acampora, the Institute of Genetics and Biophysics A. Buzzati-Traverso-CNR and Dr. Rosita Stanzione, NEUROMED I.R.C.C.S.) were extracted from the whole brain, midbrain, and cerebellum of four rats from independent litters (Sprague Dawley and Wistar stocks). For the reverse transcription, oligo-dT or random hexamer primers and Superscript II ™ retrotranscriptase (Thermo Fisher Scientific) were used, according to the manufacturer’s protocol. Nine independent cDNA preparations were obtained, including the preparation from the whole mouse brain, to check the species-specificity of PCR primers.

VAMP7-specific forward primers were designed on exons 2 and 4, whereas reverse primers were designed on exons 7 and 8 of the rat sequence (accession number NM_053531.2). Different combinations of those primers were employed in end-point PCR experiments, with the aim of amplifying even the lowest amount of the rat VAMP7j isoform. Primers labeled as rVAMP7_ex4F_2 (5′-CTCGAGAGCACAGACCGCAC-3′) and rVAMP7_ex7R_2 (5′-CAAGGTTCCTGCTGGTCGTCT-3′) were used both in simplex and nested PCR amplifications (Wonder Taq, New England Biolabs), to produce a doublet of 263 bp for VAMP7a (main isoform, used as the positive control), and a theoretical band of 104 bp for VAMP7j.

In the nested end-point PCR, pre-amplification of samples (20× cycles) with rVAMP7 _ex2F (5′-TGCCAGGGGAACCACTATTC-3′, specific for the exon 2) and rVAMP7_ex8R (5′-CACAGCTTGGCCACGTGAAG-3′, specific for the exon 8) primers pair was exploited. Then, 0.1 volume of pre-amplified samples was used as the template for a second PCR (35× cycles), employing rVAMP7_ex7R_2 and rVAMP7_ex4F_2 primers. The amplification of VAMP7a isoform was used as a loading control of the 2-step PCR experiment. An isoform-specific primer for VAMP7j was also designed at the putative junction of exons 4 and 7 of the rat sequence. It was labeled as rVAMP7j_ex4-7F (5′-CGTTCTGGCTGCACAACTGTCC-3′, wherein underlined bases belong to the 5′-end of exon 7) and used in qPCR amplifications with rVAMP7_ex7R (5′-TGACGTTCTTCACGCACATGG-3′, expected amplicon: 80 bp) or rVAMP7_ex8R (expected amplicon: 170 bp). Rat VAMP7a served as an amplification control by using rVAMP7_ex6F (5′-CAGCGTGGAGAAAGGCTAGAA-3′) and rVAMP7_ex7R primers pair (expected amplicon: 118 bp).

qPCR was performed in triplicates, using Sso Advance Universal SYBR Green supermix (Bio-Rad, Hercules, CA, USA) on CFX96 Real Time PCR system (Bio-Rad), according to the manufacturer’s protocols.

### 4.6. Western Blotting

In Western blot experiments, 200 k cells were transfected as already described either with VAMP7j, VAMP7j_45F or EGFP, harvested 24 h after transfection and resuspended in RIPA buffer with Complete EDTA-free protease inhibitor (ThermoFisher). Samples were separated by standard SDS-PAGE and blotted onto nitrocellulose filters using protean and transblot cells (Bio-Rad). Membranes were blocked in 5% defatted milk in TBS-Tween for 45 min at RT.

Anti-L1CAM (Abcam, AB24345) and anti-GAPDH (Sigma, G9545) antibodies were diluted 1:5000 in blocking solution and incubated O/N at 4 °C. Secondary antibodies (anti mouse IgG-HRP, A16066, Novex antibodies (ThermoFisher); anti-rabbit IgG-HRP, SSA004, Sino Biologicals, Beijing, China) were diluted 1:10,000 in blocking solution and incubated 2 h at R_T_. Enhanced chemiluminescence detection was performed using the Supersignal system (Pierce, Appleton, WI, USA), following the manufacturer’s instructions.

### 4.7. Cell Viability Assay

The toxicity of VAMP7j and its mutant versions was assessed with CytoTox-ONE™ Homogeneous Membrane Integrity Assay (Promega Italia, Milano, Italy). Cells were transfected in proliferative and differentiative conditions, as already described, and grown for 24 h. Culture media was then harvested, and an equal volume of CytoTox-ONE™ reagent was added and samples were incubated for 10 min. Then, the reaction was stopped with a blocking solution and fluorescence at 590 nm was detected using a Fluoroskan Ascent fluorometer (ThermoFisher). A negative (medium without cells) and positive (cells lysed with Promega Lysis Solution) control were used to determine blank and maximal LDH release. Percent cytotoxicity (% CT) was then calculated as follows:

%CT = 100 (Sample − Blank)/(Maximum_LDH_release − Blank)

### 4.8. Genome- and Transcriptome In Silico Analyses

Available entries for *SYBL1* orthologues at the GenBank and EMBL databases were considered to infer the conservation of the exon–intron structure and the chromosomal location in Metazoa. In silico screening of transcriptomic datasets (including those from the Sequence Reads Archive) was performed by discontiguous MegaBLAST at the NCBI BLAST web server (https://blast.ncbi.nlm.nih.gov/Blast.cgi), using as queries two VAMP7j subsequences of 180 bp (or 360 bp), consisting of bases −90 to + 90 (or −180 to +180), with zero position being the exon junction created by the skipping event specific to this isoform. Only experimentally determined sequences were considered as positive hits, whereas all predicted and model sequences were excluded.

### 4.9. Structure Modelling and Docking

Initial models of VAMP7j in closed conformation were obtained at the Phyre2 web server, using the one-to-one threading protocol and the crystal structure of VAMP7a bound to VARP (PDB ID 4B93) as a template [17,61]. Models of the ankyrin repeats from proteins Vps9 domain and ankyrin repeats (VARP) and leucine-rich repeats Kinase 1 (LRRK1) (residues 659–859 and 86–222, respectively) were obtained using the ColabFold implementation of Alphafold, with default settings [62].

Model quality was estimated by the QMEAN and GQME indexes at the SwissModel webserver. Finally, ModRefiner at the Zhang Lab webserver was used to refine models [63,64,65]. Models of VAMP7j_Y45p were obtained by editing the structure with CHARMM-GUI PDB reader tool, to introduce a phosphotyrosine at position 45 [66,67].

Protein–protein docking simulations were performed using ClusPro 2.0, by assigning attraction between tyrosine 45 in VAMP7j structure and regions 668–698 and 86–116 of VARP and LRRK1, respectively, as such regions are known to bind VAMP7a [68]. The resultant highest score model was used for subsequent analysis.

### 4.10. Molecular Dynamics

All-atom MD simulations were performed with Gromacs 2022.3 [69,70] using the Charmm36-jul2021 force field [71,72].

Models were solvated with the TIP3 water model in a triclinic box, with a minimum distance of 1 nm between the protein and the box surface; 0.15 M NaCl was added to simulate physiological ionic strength and neutralize the system. Energy was minimized by 5000 steps of steepest descent energy minimization, with a tolerance of 1000 kJ mol^−1^ nm^−1^. Subsequently, a 200 ps NVT MD simulation was used to heat the system from 0 to 100 K with restraints lowered to 400 kJ mol^−1^ nM^−2^. Then, the system was heated up to 310 K in 400 ps during an NPT simulation with further lowered restraint (200 kJ mol^−1^ nM^−2^). Finally, the system was equilibrated during an NPT simulation for 1 ns with backbone restraints lowered to 50 kJ mol^−1^ nM^−2^. All restraints were removed for the 1 µs production run.

The V-rescale thermostat was used to equilibrate the temperature, whereas the C-rescale barostat was used to control the pressure [73,74]. Newton’s equation of motion was integrated using a leapfrog algorithm with a 2-fs time step. The particle mesh Ewald (PME) method was used to compute the long-range electrostatic forces and H-bonds were constrained with the LINCS algorithm [75,76].

### 4.11. Umbrella Sampling

In the umbrella sampling method, a fast-pulling simulation (FPS) is performed to separate the members of a complex by applying an external force along the reaction coordinate *ξ*. In such simulations, one member of the complex is restrained, and serves as a reference, whereas the other is free to move along *ξ*.

During the simulations, the distance between the center of mass (COM) of the two species increases because of the external force that destabilizes the equilibrium conditions. The force–time curves generated in these pulling simulations are not suitable to determine the free energy of dissociation of the complex, as the dissociation is a path-dependent process. Therefore, sampling windows of 0.1–0.2 nm along the reaction coordinate are defined, and independent MD simulations are used to generate an ensemble of structures along the reaction coordinate. The potential of mean force (PMF) can be calculated as a function of the reaction coordinate by reassembling adjacent windows by the weighted histogram analysis method (WHAM). Then, the ∆G can be computed as the difference between the highest and lowest point of the PMF.

Gromacs 2022.3 was used to simulate the LRRK1-VAMP7j complex in its phosphorylated and unphosphorylated form. The Charmm36f forcefield was used to parametrize the proteins and water was simulated with a TIP3 model. The protein complex was put into a tetrahedral box with a size of 6.904 nm × 5.484 nm × 14.669 nm and the center of the model was put at 3.700 nm × 2.492 nm × 3.786 nm with respect to box origin. The system was solvated and 0.15 M NaCl was added to simulate physiological ionic strength and neutralize the system. Initial energy minimization, NVT and NPT equilibrations were performed as previously described; however, alpha carbons were restrained at every point of the simulation by applying a 1000 kJ mol^−1^ nM^−2^ restraining penalty.

The final states of the NPT simulation were used as input to perform a fast-pulling simulation (FPL) of VAMP7j from LRRK1. During such FPL simulation, VAMP7j was forced to dissociate from LRRK1 applying an external harmonic force along the reaction coordinate. Specifically, the cantilever spring constant and the pulling velocity were set at 1000 kJ mol^−1^ nM^−2^ and 0.01 nm ps^−1^, respectively. Application of slower pulling rates and forces resulted in the production of nearly identical trajectories and similar forge vs. time curves; thus, the faster pulling rate was applied to the whole system, to hasten data collection while preserving the reliability of the result.

After 500 ps of FPL simulation, the centers of mass (COM) of the two proteins were separated by a total of 5 nm. The coordinates of the solvated complex were recorded every 1 ps, and the Cα atoms of the protein complex were restrained throughout the simulation. Windows at approximately 0.2 nm from each other were selected for independent MD simulations. Specifically, each window was equilibrated in a 100 ps NPT simulation, followed by a 10 ns production run with the settings described in the previous section. The results were analyzed with the WHAM method, using 50 bins and 200 rounds of bootstrapping analysis.

### 4.12. Statistical Analysis

Data are presented as a mean ± standard deviation (mean ± SD) and statistical significance was analyzed using one-way ANOVA with Tukey’s correction (when the mean of all samples is compared to that of all the others) or with Dunnet’s correction (when the mean of all samples is only compared to a single control).

## Figures and Tables

**Table 1:** List of antibodies (Ab) used for immunofluorescence experiments in this work. Producer and batch details are reported.

Ab	Brand	Serial
GFP	ThermoFisher	A6455
L1CAM	ABCAM	AB24345
LRRK1	Abnova	H00079705-M03

## Data Availability

Not applicable.

## Supplementary Materials

The supporting information can be downloaded at: https://www.mdpi.com/article/10.3390/ijms242417326/s1.
