# Supplementary material for: VAMP7j: A Splice Variant of Human VAMP7 That Modulates Neurite Outgrowth by Regulating L1CAM Transport to the Plasma Membrane
Source: Int J Mol Sci. 2023 Dec 10;24(24):17326. doi: 10.3390/ijms242417326 (PMC10743575; doi:10.3390/ijms242417326)
Supplement: Supplementary file 1 [file ijms-24-17326-s001.zip › ijms-2718961-supplementary.pdf]

Supplementary information to manuscript:

VAMP7j: a splice variant of human VAMP7 that modulates neurite outgrowth by regulating L1CAM transport to the plasma membrane

Matteo Gasparotto [1], Elena Dall'Ara [1], Marcella Vacca [2,*] and Francesco Filippini [1,*]

[1] Synthetic Biology and Biotechnology Unit, Department of Biology, University of Padua, via U. Bassi 58/B, 35131 Padova, Italy; matteo.gasparotto.1@phd.unipd.it (M.G.); elena.dallara@studenti.unipd.it (E.D.); francesco.filippini@unipd.it (F.F.)
[2] Institute of Genetics and Biophysics "A. Buzzati Traverso", CNR, via Pietro Castellino, 111, 80131, Naples, Italy; marcella.vacca@igb.cnr.it
* Correspondence: marcella.vacca@igb.cnr.it (M.V.); francesco.filippini@unipd.it (F.F.)

	CNS	Liver	Intest.	Plac.	Lung	Adren.	Stom.	Kidney	Testis	Ovary	Heart	Muscle
Homo sapiens	+	+	-	+	-	+	+	+	-	+	+	-
Haplorrhini	+	+	+	-	+	-	-	+	+	-	-	-
Platyrrhini	+	-	-	-	-	-	-	-	-	-	-	-
Lagomorpha	-	-	-	-	-	-	-	+	-	-	+	+
Rodentia	-	-	-	-	-	-	-	-	-	-	-	-

Supplementary Table 1. VAMP7j expression in mammals (in silico evidence from Sequence Read Archive). Among primates, Haplorrhini include species representative for hominoidea (*Pan troglodytes*), old world (*Macaca mulatta* and *Chloracebus sabaeus*) and new world (*Callithrix jaccus*) monkeys. Platyrrhini include lemuriformes (*Microcebus murinus* and *Daubentonia madascariensis*) and lorisiformes (*Otolemur garnettii*). Lagomorpha are represented by *Oryctolagus cuniculus*, while Rodentia include *Rattus norvegicus* and *Mus musculus*. Abbreviations: CNS, central nervous system; Intest., intestine; Plac., placenta; Adren., adrenal tissue; Stom., stomach. Symbols: "+" is reported where one or more positive hit is found, whereas "-" just means "not found" while not being "evidence of absence", because of the partial coverage of each organism dataset. No positive hits were found in adipose tissue, spleen, colon, and pancreas and hence these tissues have no corresponding columns in the table to save space.

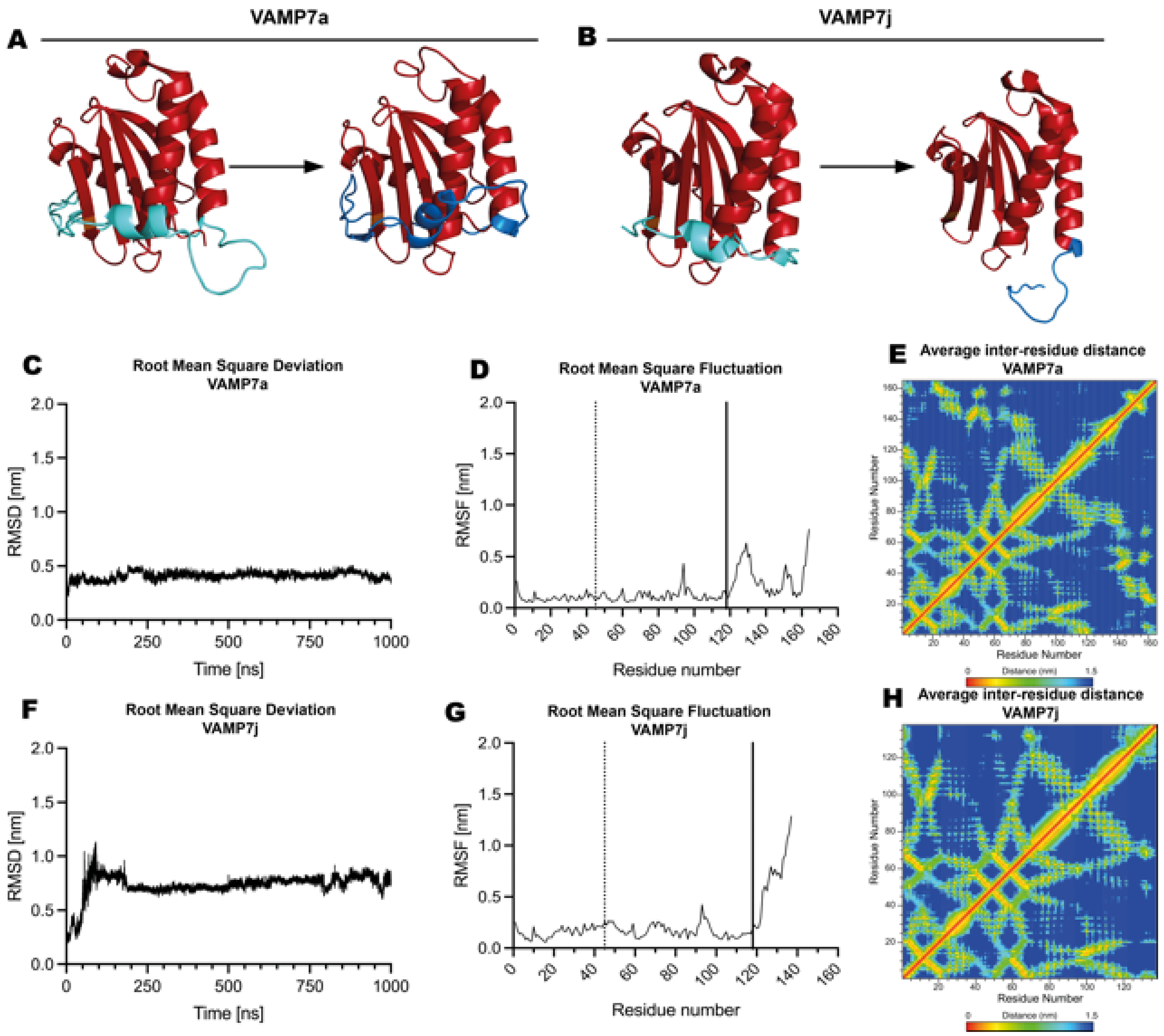

Supplementary Figure 1. Molecular dynamics simulations predict VAMP7j does not adopt a closed conformation. (**A-B**) Models of VAMP7 compared to the average structure adopted during the molecular dynamics simulation. While VAMP7a SNARE motif (light blue, initial status; dark blue, final status) remains bound to the LD (red) for the whole length of the simulation, the average structures for the VAMP7j simulation indicates a strong displacement of VAMP7j $SNARE_{C\text{-}ter}$ from the original position. (**C-D**) RMSD between the original structure and each frame of the molecular dynamics simulation. Simulations of VAMP7a rapidly reach convergence, with RMSD values stabilizing around 0.5 nm; conversely, VAMP7j simulations show higher variation, due to movement of the $SNARE_{C\text{-}ter}$. (**E-F**) RMSF of each residue from the average structure adopted by VAMP7 during the simulation. The dashed line indicates Y45, whereas the black one indicates the boundary between the LD (residues 1-118) and the $SNARE_{C\text{-}ter}$. Longin domain appear stable in all simulations, whereas the SNARE motif has a higher mobility, which is highly increased in simulation of VAMP7j. (**G-H**) Average inter-residue distance during the simulations. Closer residues are depicted in red, whereas residues at a distance equal or higher of 1.5 nm are shown in blue. Maps indicate only transient contacts between the longing domain and the SNARE motif of VAMP7j.

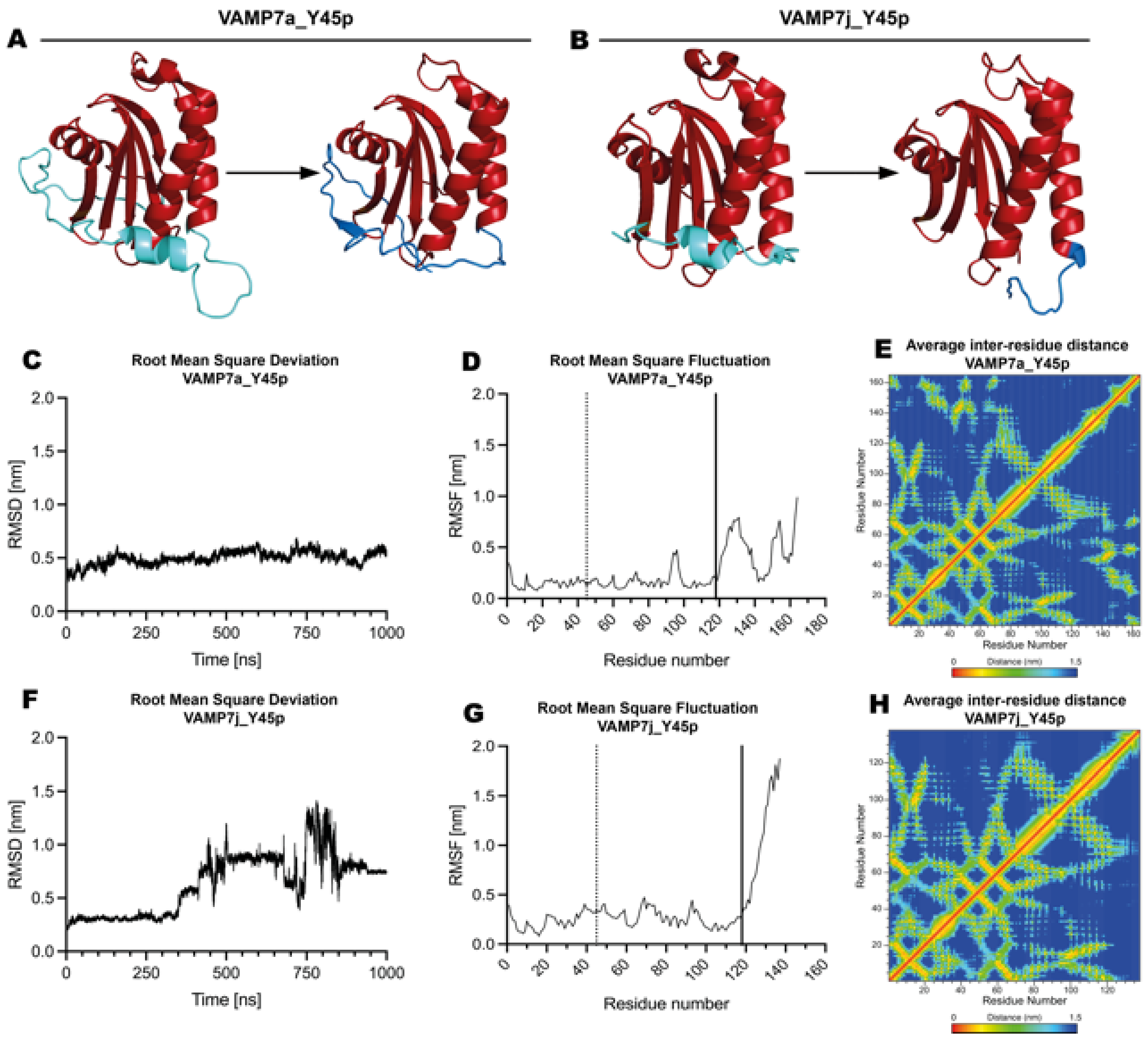

Supplementary Figure 2. Molecular dynamics simulations predict Y45 phosphorylated VAMP7j does not adopt a closed conformation. Panel descriptions are the same as in caption for Supplementary Figure 1, but in this figure, they concern the Y45 phosphorylated counterparts.

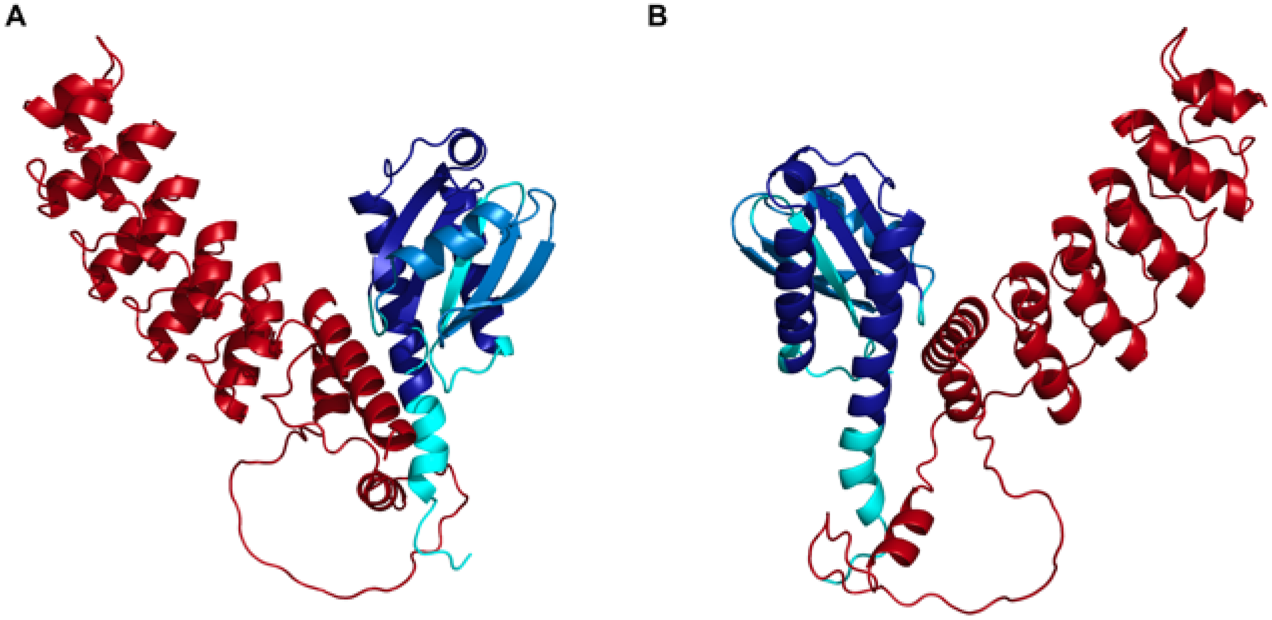

Supplementary Figure 3. Docked model of VAMP7j bound to VARP. (**A**) front view and (**B**) back view. VARP (red) does not interact with any of VAMP7 region known to mediate protein-protein interaction (blue). Contacts are only established with the extreme C-termini of the SNARE motif and the last portion of the α_Y region of the Longin domain (darker blue).
